# Elevated blood pressure is associated with higher prevalence of low visual acuity among adolescent males in Northeast China

**DOI:** 10.1038/s41598-017-14252-9

**Published:** 2017-11-22

**Authors:** Miaomiao Zhao, Wei Wang, Han Yu, Yunsheng Ma, Liqiang Zheng, Lijuan Zhang, Guiping Wu, Yingxian Sun, Jue Li

**Affiliations:** 1 Key Laboratory of Arrhythmias of Ministry of Education of China, Tongji University School of Medicine, Shanghai, China; 2 Institute of Clinical Epidemiology, Tongji University School of Medicine, Shanghai, China; 3 Division of Preventive and Behavioral Medicine, Department of Medicine, University of Massachusetts Medical School, Worcester, MA USA; 4 Department of Clinical Epidemiology, Library, Shengjing Hospital of China Medical University, Shenyang, Liaoning China; 5 Department of Cardiology, the Second Hospital Affiliated to Shenyang Medical College, Shenyang, Liaoning China; 6 Department of Cardiology, the First Hospital of China Medical University, Shenyang, Liaoning China

## Abstract

The purpose of this study is to track the trends of low visual acuity (VA) from 2005 to 2014, and to investigate its associations with systemic blood pressure (BP) components among adolescents in Northeast China. A total of 55320 students of Han nationality aged 13 to 18 years were included. There has been a significant increase in the prevalence of low VA, with 31.3% in 2005, 40.2% in 2010 and 43.4% in 2014. In multivariable-adjusted logistic regression models, each 1-mm Hg increment in systolic BP (SBP) was associated with 0.8% (95% confidence interval [CI]: 0.1–1.6%), 0.5% (95% CI: 0.1–0.9%) and 1.1% (95% CI: 0.6–1.6%) increased odds of low VA for males in 2005, 2010 and 2014; each 1-mm Hg increment in pulse pressure (PP) was associated with 1.6% (95% CI: 0.7–2.5%), 0.8% (95% CI: 0.4–1.2%) and 1.2% (95% CI: 0.7–1.7%) increased odds of low VA. Higher PP categories had greater odds for low VA compared with the reference group. Similar associations were not observed for females. We conclude that higher prevalence of low VA was significantly associated with higher SBP and PP in males. Furthermore, there was a dose-dependent association between the prevalence of low VA and the levels of PP.

## Introduction

Low visual acuity (VA) in school-age children is a major public health problem worldwide^1–4^. The World Health Organization (WHO) has identified low VA as one of the five areas of priority in VISION 2020, especially school-age children in developing countries^5^. Low VA can negatively affect children’s neurological development^6^ and quality of life^7^. Additionally, childhood vision loss imposes a heavy socioeconomic burden on individuals, families, and society^8–10^. In mainland China, the prevalence of low VA among children and adolescents has increased rapidly since 1985. The projected number of children and adolescents aged 7 to 18 years with low vision has been estimated to rise to 152 million by the year 2020, and 180 million by the year 2030^11^.

Hypertension is one of the leading causes of cardiovascular disease, which contributes to more than seven million deaths each year worldwide^12^. Pathophysiological and epidemiological studies have demonstrated that elevated blood pressure (BP) during childhood is a strong predictor for hypertension in adulthood^13,14^. Furthermore, children with elevated BP also have an increased risk of developing end-organ damage, such as ventricular hypertrophy and increased carotid intima-media thickness^15,16^. There has been a remarkable increase in BP levels and prevalence of hypertension among children and adolescents over the past 30 years, not only in China, but also in other developed countries^17–22^.

Due to the dramatic increases in childhood low VA and hypertension, it is possible that these health issues might have some degree of interaction or correlation. Systemic BP has received significant attention for its role in the development and progression of various age-related ocular diseases. Epidemiological studies have indicated the association of BP components with glaucoma^23,24^, cataract^25,26^ and macular degeneration^27,28^ in the middle aged and elderly. However, to our knowledge, there has been no research into the associations between low VA and systemic BP components in children and adolescents.

Liaoning Province is located in northeast China, with an average population density of 297 individuals per square kilometer, and is ranked 8th in total gross domestic product (GDP) in China. On the basis of three large cross-sectional surveys between 2005 and 2014 among high-school students from Liaoning Province, China, the purpose of the present study was to track the trends of low VA during the past decade, and to further investigate its associations with systemic BP components in adolescents in Northeast China.

## Methods

### Study population

Methods used in the present study were carried out in accordance with the approved guideline during research for human subject protection. At the same time, all experimental protocols were approved by the Ethics Committee of Tongji University, and informed consent was obtained from all students and their parents.

Data were obtained from three successively cross-sectional surveys carried out by the government in 2005, 2010, and 2014 in Liaoning province, China, which investigated the health status in Chinese school-age children. The surveys used a stratified multistage sampling method to randomly select schools from 14 cities in Liaoning Province (Shenyang, Dalian, Anshan, Fushun, Benxi, Dandong, Fuxin, Jinzhou, Yingkou, Liaoyang, Panjin, Tieling, Chaoyang, and Huludao). The sample size in each of the 14 cities was equal. All subjects were either primary or secondary school students. To ensure the accuracy of the survey comparisons conducted across 2005, 2010 and 2014, students were selected from the same urban and rural areas, where over 85% of the sampled schools remained the same across the different years. At the same time, the sampling strategies, measurements and quality control of all three surveys were identical.

In the present study, only junior and senior high school students of Han nationality, aged 13 to 18 years were included. Of the 55709 participants, we excluded 389 (0.7%) students due to missing data or extreme height, weight and BP values (>5 SDs from the survey year; sex- and age-specific mean). Finally, a total of 55320 participants (19265 in 2005, 19173 in 2010 and 16882 in 2014) with complete records on age, sex, region of habitation, height, weight, BPs, and VA data were included for further analyses. The distributions for sex and region were similar throughout the surveys (*P* = 0.875 and *P* = 0.279), with an approximate ratio of 1:1 in each survey year (see Supplementary Table S1).

### VA measurements

Unaided distance VA was measured for the right eye, then the left eye, using a retro-illuminated Log MAR chart with tumbling-E optotypes (Precision Vision) in rooms with an illumination of approximately 500 lux. Students were required to indicate the direction of the E optotype within 5 seconds, and were observed closely for any squinting. Measurements began at a distance of 5 m, starting from the fourth line from the bottom (20/20). If the student could identify the optotype, the optometrist pointed to the next smaller line. If he/she failed, the optometrist pointed to the next bigger line. The lowest line correctly read determined the VA for the eye. If the top line was not read correctly, participants were advanced to 2.5 m, and subsequently to 1 m^1,11,29^. VA examinations were performed by certified optometrists using a uniform protocol throughout all surveys. Because of limited resources, presenting VA and best-corrected VA were not measured. Low VA was defined as VA worse than 20/70.

### BP measurements

BP was measured according to the recommendations by the National High Blood Pressure Education Program (NHBPEP) Working Group in Children and Adolescents^30^ using an auscultation mercury sphygmomanometer with an appropriate cuff size for students. The stethoscope was placed over the brachial artery pulse, proximal and medial to the cubital fossa and below the bottom edge of the cuff. Appropriately sized cuffs were used to cover at least 40% of the arm circumference at a point midway between the olecranon and the acromion of the right arm. Students were placed in a sitting position and allowed to rest for at least 5 min prior to measurement of BP. Systolic BP (SBP) was defined as the onset of “tapping” Korotk off sounds (K1), while diastolic BP (DBP) was defined as the fifth Korotk off sounds (K5). The mean of three separate BP measurements were calculated for each student. Pulse pressure (PP) was calculated as the systolic minus diastolic pressure.

### Covariates

We included demographic variables (age, sex, region of habitation), body mass index (BMI), and behavioral variables (sleep duration, outdoor activity time, and homework time). Anthropometric measurements were conducted according to the same protocol by the same technicians in each administrative region across all surveys. Students were asked to wear light clothes, and stand straight on bare feet. Both height and weight were measured twice, and the mean value was recorded. BMI was calculated according to the weight divided by height squared (kg/m^2^). In 2010 and 2014, all students were asked to fill out a questionnaire regarding various behavioral factors. In 2005, the questionnaire was conducted in some of the students who had received physical examinations (5514 out of 19265). There were no significant differences in demographic variables, BMI, BP values, and prevalence of low VA between students who undertook the questionnaire and those who did not for both sexes. In this paper, we used the following questions: “How many hours per day do you sleep?” (<6 h, 6–7 h, 7–8 h, 8–9 h, 9–10 h, ≥10 h); “How many hours per day do you do outdoor activity?” (<0.5 h, 0.5–1 h, 1–2 h, ≥ 2 h); “How many hours per day do you do your homework?” (<0.5 h, 0.5–1 h, 1–2 h, 2–3 h, ≥3 h). In the subsequent analyses, the categories were translated to time as these specific values: <0.5 h = 0.25 h, 0.5–1 h = 0.75 h, 1–2 h = 1.5 h, ≥2 h = 2.5 h, 2–3 h = 2.5 h, ≥3 h = 3.5 h, <6 h = 5.5 h, 6–7 h = 6.5 h, 7–8 h = 7.5 h, 8–9 h = 8.5 h, 9–10 h = 9.5 h, ≥10 h = 10.5 h.

### Statistical analyses

All analyses were performed separately for both sexes. Raw prevalence of low VA and standardized prevalence of low VA based on the age distribution in 2005 were estimated in different survey years. Chi-squared test was used to evaluate the differences in prevalence between two adjacent years. We reported the characteristics of adolescents in 2005, 2010 and 2014, and compared them between different sex groups. Associated factors of low VA were also compared between normal VA group and low VA group. Continuous variables were summarized as mean ± SD, and the comparison between groups was based on student’s t-test; categorical variables were presented as percentage and the comparison between groups were based on Chi-squared test. Multivariable logistic regression analyses were conducted to evaluate associations of low VA with BP components adjusted for covariates. SBP, DBP and PP were entered into the multivariable models separately. We constructed 3 models: in Model 1, we adjusted for age and region of habitation; in Model 2, we adjusted for age, region of habitation and BMI; and in Model 3, we adjusted for age, region of habitation, BMI, sleep duration, outdoor activity time and homework time. PP values were also categorized by 10 mm Hg increments to study the association with low VA in multivariable models, using the lowest category as the reference group (≤30 mm Hg). All analyses were performed using SPSS statistical software version 20.0 (SPSS Inc., Chicago, IL). Two-side *P* values less than 0.05 indicated statistical significance.

## Results

### Trends of low VA

The raw and standardized prevalence of low VA for each survey year based on sex and region of habitation were presented in Table 1. There has been a significant increase in the prevalence of low VA over the past decade, with 31.3% in 2005, 40.2% in 2010 and upwards of 43.4% in 2014. A similar pattern was observed in both sexes and all subgroups during the ten-year period (all *P* < 0.001 between survey years). Additionally, low VA was more prevalent in females than males; and in urban areas than in rural areas at different time points (all *P* < 0.001).

**Table 1:** Raw and standardized prevalence of low VA among adolescents from 2005 to 2014^a^.

Classification	2005				2010				2014			
	n	N	%, Raw	%, Standardized	n	N	%, Raw	%, Standardized	n	N	%, Raw	%, Standardized
Overall male	2646	9657	27.4	27.4	3335	9572	34.8	34.8**	3287	8471	38.8	39.1**
Urban male	1524	4829	31.6	31.6	1928	4700	41.0	40.8**	2009	4257	47.2	47.3**
Rural male	1122	4828	23.2	23.2	1407	4872	28.9	28.9**	1278	4214	30.3	30.7**
Overall female	3375	9608	35.1	35.1	4374	9601	45.6	45.5**	4013	8411	47.7	47.8**
Urban female	1924	4827	39.9	39.9	2507	4754	52.7	52.6**	2274	4145	54.9	54.9**
Rural female	1451	4781	30.3	30.3	1867	4847	38.5	38.6**	1739	4266	40.8	40.9**
Total	6021	19265	31.3	31.3	7709	19173	40.2	40.2**	7300	16882	43.2	43.4**

Abbreviations: VA, visual acuity. ^**a**^Difference of prevalences between two adjacent years were examined by Chi-squared test, **P < 0.001.

### Participants’ Characteristics

The general characteristics of the participants stratified by sex of the three surveys were shown in Table 2. Overall, males had higher levels of BMI, SBP, DBP, PP and a greater percentage of high PP levels than females. Male students also had longer sleep duration, more time spent for outdoor activity, and less time spent for homework than females (all *P* < 0.05). There were no significant differences in age distribution and region proportion between males and females.

**Table 2:** Participant characteristics and associated factors for low VA.

Characteristics	2005			2010			2014		
	Males (N = 9657)	Females (N = 9608)	*P* value	Males (N = 9572)	Females (N = 9601)	*P* value	Males (N = 8471)	Females (N = 8411)	*P* value
Age, year	15.51 ± 1.71	15.51 ± 1.71	0.987	15.52 ± 1.70	15.52 ± 1.70	0.871	15.44 ± 1.70	15.47 ± 1.70	0.283
Urban, n (%)	4829(50.0)	4827(50.2)	0.745	4700(49.1)	4754(49.5)	0.566	4257(50.3)	4145(49.3)	0.206
BMI, kg/m^2^	20.06 ± 3.28	19.81 ± 2.84	<0.001	20.68 ± 3.42	20.27 ± 2.95	<0.001	21.26 ± 3.89	20.47 ± 3.21	<0.001
SBP, mm Hg	111.67 ± 12.77	105.86 ± 11.32	<0.001	113.20 ± 12.21	106.69 ± 11.32	<0.001	116.73 ± 10.91	110.42 ± 10.44	<0.001
DBP, mm Hg	70.47 ± 8.88	68.47 ± 8.28	<0.001	68.71 ± 10.64	66.98 ± 9.80	<0.001	72.91 ± 8.60	70.34 ± 8.62	<0.001
PP, mm Hg	41.20 ± 9.91	37.39 ± 8.30	<0.001	44.49 ± 11.21	39.71 ± 9.88	<0.001	43.81 ± 9.92	40.08 ± 8.34	<0.001
PP categories, n (%)									
≤30	2125(22.0)	3234(33.7)	<0.001	1429(14.9)	2627(27.4)	<0.001	877(10.4)	1471(17.5)	<0.001
31–40	3990(41.3)	4241(44.1)		3282(34.3)	3738(38.9)		3579(42.3)	4173(49.6)	
41–50	2365(24.5)	1672(17.4)		2733(28.6)	2288(23.8)		2477(29.2)	2085(24.8)	
≥51	1177(12.2)	461(4.8)		2128(22.2)	948(9.9)		1538(18.2)	682(8.1)	
Sleep duration, h/day	7.30 ± 1.10^#^	7.23 ± 1.01^#^	0.015^#^	7.29 ± 1.06	7.25 ± 1.00	0.005	7.30 ± 1.07	7.23 ± 1.00	<0.001
Outdoor activity time, h/day	0.93 ± 0.58^#^	0.82 ± 0.50^#^	<0.001^#^	0.86 ± 0.56	0.72 ± 0.50	<0.001	0.96 ± 0.60	0.84 ± 0.52	<0.001
Homework time, h/day	1.73 ± 1.02^#^	1.89 ± 0.99^#^	<0.001^#^	1.59 ± 0.96	1.72 ± 0.96	<0.001	1.81 ± 1.02	1.99 ± 0.98	<0.001

Abbreviations: VA, visual acuity; BMI, body mass index; SBP, systolic blood pressure; DBP, diastolic blood pressure; PP, pulse pressure. ^#^Data available for participants who undertook questionnaire.

### Comparisons of associated factors between normal VA and low VA group

As shown in Table 3, participants in low VA group had older age, higher prevalence of urban region of habitation, higher BMI, shorter sleep duration, and less time spent for outdoor activity compared to those in normal VA group for both sexes in all three surveys, and additionally had more time spent for homework for both sexes in 2005 and 2014 (all *P* < 0.05). For males, participants in low VA group had higher DBP in 2005 and 2014, and higher SBP and PP in 2005, 2010 and 2014 (all *P* < 0.01). For females, participants in low VA group had higher DBP in 2005, and higher SBP and PP in 2005 and 2014 (all *P* < 0.05).

**Table 3:** Comparisons of associated factors between normal VA and low VA group.

Characteristics	2005			2010			2014		
	Normal VA	Low VA	*P* value	Normal VA	Low VA	*P* value	Normal VA	Low VA	*P* value
**Males**	N = 7011	N = 2646		N = 6237	N = 3335		N = 5184	N = 3287	
Age, year	15.26 ± 1.70	16.16 ± 1.55	<0.001	15.30 ± 1.70	15.93 ± 1.62	<0.001	15.24 ± 1.70	15.75 ± 1.65	<0.001
Urban, n (%)	3305(47.1)	1524(57.6)	<0.001	2772(44.4)	1928(57.8)	<0.001	2248(43.4)	2009(61.1)	<0.001
BMI, kg/m^2^	19.92 ± 3.22	20.44 ± 3.40	<0.001	20.63 ± 3.47	20.79 ± 3.33	0.030	21.19 ± 3.88	21.38 ± 3.90	<0.026
SBP, mm Hg	110.97 ± 12.76	113.53 ± 12.61	<0.001	112.79 ± 12.25	113.96 ± 12.08	<0.001	115.90 ± 11.08	118.03 ± 10.50	<0.001
DBP, mm Hg	70.22 ± 8.86	71.12 ± 8.88	<0.001	68.65 ± 10.60	68.81 ± 10.70	0.464	72.71 ± 8.53	73.23 ± 8.70	0.006
PP, mm Hg	40.75 ± 9.77	42.41 ± 10.18	<0.001	44.14 ± 11.31	45.14 ± 10.98	<0.001	43.19 ± 9.85	44.80 ± 9.94	<0.001
Sleep duration, h/day	7.37 ± 1.11^#^	7.13 ± 1.07^#^	<0.001^#^	7.36 ± 1.08	7.16 ± 1.03	<0.001	7.38 ± 1.09	7.19 ± 1.02	<0.001
Outdoor activity time, h/day	0.94 ± 0.58^#^	0.89 ± 0.57^#^	0.039^#^	0.88 ± 0.57	0.81 ± 0.54	<0.001	0.99 ± 0.61	0.92 ± 0.58	<0.001
Homework time, h/day	1.67 ± 1.01^#^	1.88 ± 1.03^#^	<0.001^#^	1.58 ± 0.96	1.60 ± 0.97	0.225	1.75 ± 1.01	1.90 ± 1.03	<0.001
**Females**	N = 6233	N = 3375		N = 5227	N = 4374		N = 4398	N = 4013	
Age, year	15.19 ± 1.69	16.09 ± 1.58	<0.001	15.21 ± 1.68	15.88 ± 1.65	<0.001	15.26 ± 1.70	15.69 ± 1.67	<0.001
Urban, n (%)	2903(46.6)	1924(57.0)	<0.001	2247(43.0)	2507(57.3)	<0.001	1871(42.5)	2274(56.7)	<0.001
BMI, kg/m^2^	19.71 ± 2.86	19.99 ± 2.80	<0.001	20.17 ± 2.98	20.38 ± 2.90	<0.001	20.33 ± 3.10	20.62 ± 3.33	<0.001
SBP, mm Hg	105.55 ± 11.30	106.43 ± 11.33	<0.001	106.55 ± 11.47	106.86 ± 11.13	0.172	110.07 ± 10.39	110.80 ± 10.49	0.001
DBP, mm Hg	68.30 ± 8.19	68.78 ± 8.42	0.007	66.86 ± 10.04	67.13 ± 9.50	0.177	70.50 ± 8.56	70.18 ± 8.69	0.091
PP, mm Hg	37.25 ± 8.32	37.65 ± 8.25	0.024	39.69 ± 10.08	39.73 ± 9.64	0.816	39.58 ± 8.23	40.63 ± 8.43	<0.001
Sleep duration, h/day	7.32 ± 1.03^#^	7.06 ± 0.96^#^	<0.001^#^	7.35 ± 1.02	7.14 ± 0.96	<0.001	7.34 ± 1.04	7.10 ± 0.95	<0.001
Outdoor activity time, h/day	0.84 ± 0.51^#^	0.77 ± 0.49^#^	<0.001^#^	0.74 ± 0.50	0.70 ± 0.50	0.001	0.87 ± 0.53	0.80 ± 0.51	<0.001
Homework time, h/day	1.84 ± 0.99^#^	1.98 ± 0.99^#^	<0.001^#^	1.71 ± 0.95	1.72 ± 0.97	0.752	1.93 ± 0.98	2.06 ± 0.98	<0.001

Abbreviations: VA, visual acuity; BMI, body mass index; SBP, systolic blood pressure; DBP, diastolic blood pressure; PP, pulse pressure. ^#^Data available for participants who undertook questionnaire.

### The association of low VA with BP components in multivariable logistic regression analyses

The associations between the prevalence of low VA and individual BP components in multivariable logistic regression models were demonstrated in Table 4. For males, higher prevalence of low VA was significantly associated with higher SBP and PP after adjusting for age, region of habitation, BMI, sleep duration, outdoor activity time and homework time in Model 3, in 2010 and 2014. Each 1-mm Hg increment in SBP was associated with 0.5% (95% confidence interval [CI]: 0.1–0.9%) and 1.1% (95% CI: 0.6–1.6%) increased odds of low VA in 2010 and 2014, respectively. Each 1-mm Hg increment in PP was associated with 0.8% (95% CI: 0.4–1.2%) and 1.2% (95% CI: 0.7–1.7%) increased odds of low VA in 2010 and 2014, respectively. For males who undertook questionnaire in 2005, higher prevalence of low VA was significantly associated with higher SBP and PP after adjusting for the potential confounders in Model 3. Each 1-mm Hg increment in SBP was associated with 0.8% (95% CI: 0.1–1.6%) increased odds of low VA. Each 1-mm Hg increment in PP was associated with 1.6% (95% CI: 0.7–2.5%) increased odds of low VA. For females, after adjusting for the potential covariates in Model 3, the prevalence of low VA was significantly associated with PP in 2014 only (OR: 1.012, 95% CI: 1.007–1.018). This adjustment resulted in a significant negative association between the prevalence of low VA and DBP in 2014, whereas there was no statistical significance using the univariate analyses, suggesting the possibility of confounding effects or effects of collineation. The odds ratios of the other variables adjusted in multivariable logistic regression model were presented in the supplementary materials (see Supplementary Table S2).

**Table 4:** Multiple logistic regression analyses of associations between low VA and BP components.

BP components	2005		2010		2014	
	Adjusted OR (95%CI)	*P* value	Adjusted OR (95%CI)	*P* value	Adjusted OR (95%CI)	*P* value
**Males**						
**SBP (per 1** **mm Hg)**						
Model 1^*^	1.004(1.000–1.008)	0.031	1.002(0.999–1.006)	0.227	1.010(1.005–1.014)	<0.001
Model 2^†^	1.004(1.000–1.008)	0.048	1.004(1.000–1.007)	0.056	1.011(1.007–1.016)	<0.001
Model 3^‡^	1.008(1.001–1.016) ^#^	0.028^#^	1.005(1.001–1.009)	0.017	1.011(1.006–1.016)	<0.001
**DBP (per 1 mm Hg)**						
Model 1^*^	0.996(0.990–1.001)	0.120	0.996(0.992–1.000)	0.044	1.000(0.995–1.006)	0.912
Model 2^†^	0.995(0.990–1.001)	0.081	0.996(0.992–1.001)	0.086	1.001(0.995–1.006)	0.809
Model 3^‡^	0.995(0.985–1.005) ^#^	0.333^#^	0.997(0.992–1.001)	0.105	1.000(0.994–1.005)	0.905
**PP (per 1 mm Hg)**						
Model 1^*^	1.010(1.005–1.014)	<0.001	1.006(1.002–1.010)	0.001	1.011(1.006–1.016)	<0.001
Model 2^†^	1.009(1.005–1.014)	<0.001	1.007(1.003–1.011)	<0.001	1.012(1.007–1.016)	<0.001
Model 3^‡^	1.016(1.007–1.025) ^#^	<0.001^#^	1.008(1.004–1.012)	<0.001	1.012(1.007–1.017)	<0.001
**Females**						
**SBP (per 1 mm Hg)**						
Model 1^*^	1.001(0.997–1.005)	0.741	1.001(0.998–1.005)	0.494	1.003(0.999–1.008)	0.115
Model 2^†^	1.001(0.997–1.005)	0.618	1.001(0.997–1.005)	0.511	1.002(0.998–1.007)	0.320
Model 3^‡^	1.005(0.998–1.013) ^#^	0.168^#^	1.001(0.997–1.005)	0.490	1.002(0.997–1.006)	0.502
**DBP (per 1 mm Hg)**						
Model 1^*^	0.999(0.993–1.004)	0.621	1.001(0.997–1.005)	0.689	0.993(0.988–0.998)	0.009
Model 2^†^	0.999(0.994–1.004)	0.708	1.001(0.997–1.005)	0.707	0.992(0.987–0.997)	0.003
Model 3^‡^	1.000(0.990–1.010) ^#^	0.969^#^	1.001(0.997–1.006)	0.569	0.991(0.986–0.996)	0.001
**PP (per 1 mm Hg)**						
Model 1^*^	1.003(0.997–1.008)	0.350	1.001(0.997–1.005)	0.701	1.013(1.007–1.018)	<0.001
Model 2^†^	1.003(0.997–1.008)	0.303	1.001(0.997–1.005)	0.718	1.012(1.006–1.017)	<0.001
Model 3^‡^	1.009(0.999–1.019) ^#^	0.066^#^	1.000(0.996–1.005)	0.839	1.012(1.007–1.018)	<0.001

Abbreviations: VA, visual acuity; BP, blood pressure; SBP, systolic blood pressure; DBP, diastolic blood pressure; PP, pulse pressure. ^*^Model 1: adjusted for age and region of habitation. ^†^Model 2: adjusted for age, region of habitation and body mass index. ^‡^Model 3: adjusted for age, region of habitation, body mass index, sleep duration, outdoor activity time and homework time. ^#^Data available for participants who undertook questionnaire.

### The association of low VA with PP categories

**Figure 1:**
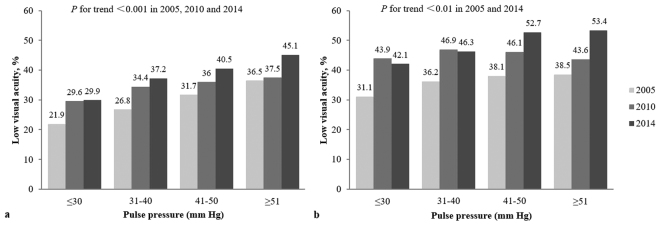
Prevalence of low visual acuity across pulse pressure categories for males (**a**) and females (**b**) (Data in 2005 available for participants who undertook questionnaire).

**Figure 2:**
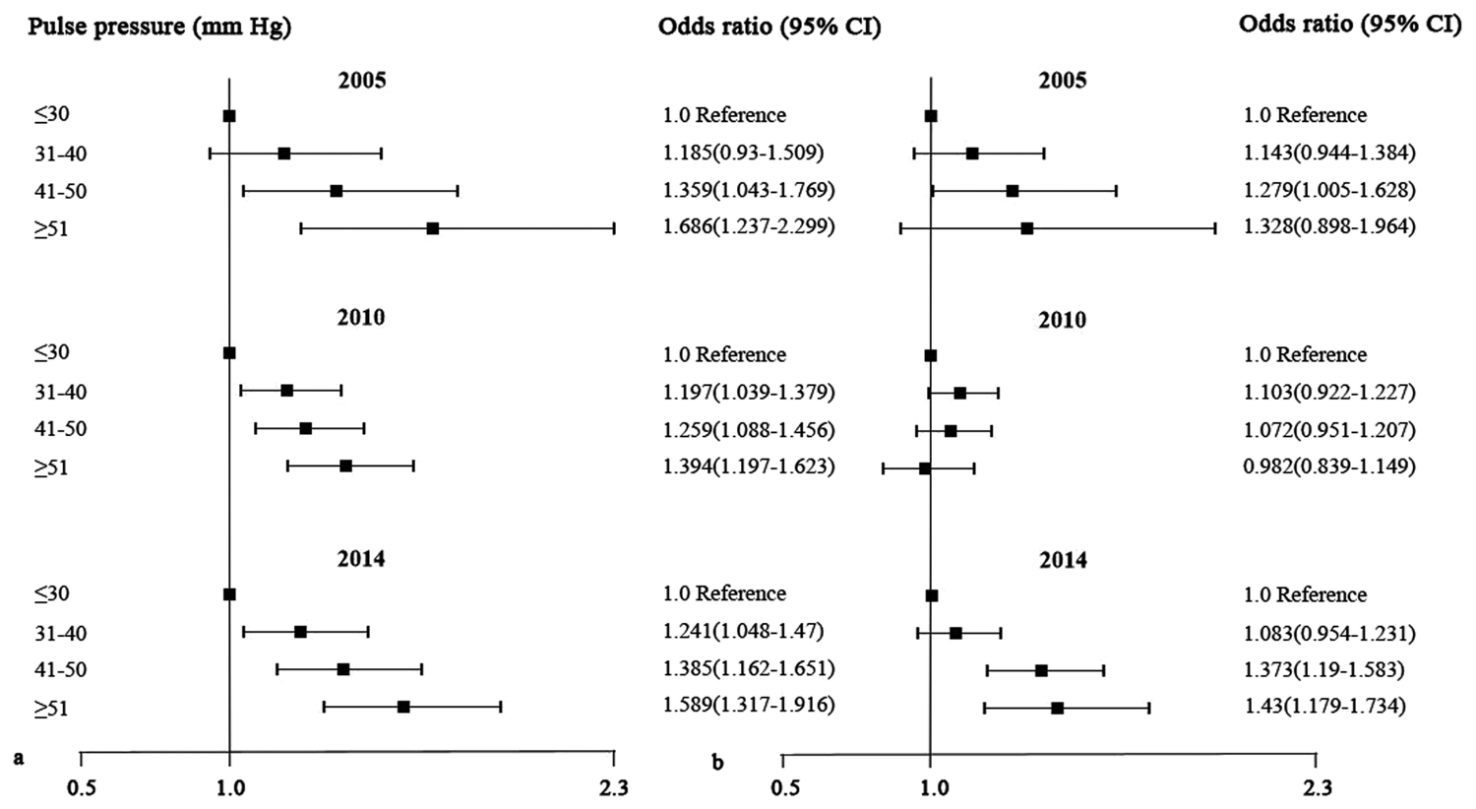
Adjusted association of low visual acuity with pulse pressure categories for males (**a**) and females (**b**) (Data in 2005 available for participants who undertook questionnaire).

We also categorized PP values by 10 mm Hg increments, which showed that the prevalence of low VA was significantly associated with increasing PP levels for males in 2005, 2010 and 2014 and females in 2005 and 2014 (all *P* for trend < 0.01, Fig. 1). In the multivariable-adjusted models, compared with the reference group (≤30 mm Hg), males with higher PP categories had greater odds for low VA after adjusting for age, region of habitation, BMI, sleep duration, outdoor activity time and homework time. However, similar results were not observed for females in all three surveys (Fig. 2).

## Discussion

The present study demonstrated the significantly increasing trends of low VA among adolescents from 2005 to 2014 in Northeast China. Our main finding demonstrated that there were significant positive associations between the prevalence of low VA with SBP and PP in males. Furthermore, prevalence of low VA showed a dose-dependent association with PP categories, that is, males with higher PP levels experienced greater prevalence of low VA.

Liaoning Province has sustained rapid economic growth in recent years. Understanding the prevalence of low VA among school children in these rapidly developing societies and encouraging early corrective measures are essential in preventing long-term visual disability and providing early warning for an epidemic of low VA. In our study, the standardized prevalence of low VA among school adolescents aged 13–18 years rose significantly over the past decade, from 31.3% in 2005, 40.2% in 2010, to 43.4% in 2014. The school system in China has become more and more competitive in recent years, with an emphasis on early educational achievements and passing examinations. Participants in our study were all junior and senior high school students, who were under significant pressure in the preparation for senior high school entrance examination (“Zhongkao”) and college entrance examination (“Gaokao”). The increased time spent engaged in visual tasks each day and decreased time spent playing outdoors, may be an important factor resulting in the increasing trends of low VA in adolescents.

The prevalence of low VA in children varies across different regions of the world and these statistics are often related to social, economic and cultural factors^9^. Additionally, excessive eye usage (such as Internet and television)^31,32^, insufficient outdoor physical activities^29,33–35^, and poor diet^31^ have been demonstrated to be the most important modifiable risk factors related to the onset of low VA and its progression. However, there is substantial interest in identifying other potentially modifiable related factors.

In this present study, we found that higher prevalence of low VA was positively related to higher SBP and PP levels in adolescent males. To our knowledge, our study is the first which investigated the association between low VA and systemic BP components among children and adolescents, and the mechanisms are still unclear. We examined the pathological mechanisms of glaucoma for better interpretation of our results. The increase in BP leads to hemodynamic alterations, either locally or systemically, resulting in the reduction of ocular blood flow through the small and medium sized blood vessels in the eye, mainly the ophthalmic artery and capillaries^36,37^. Current evidence suggests that ocular blood flow reduction contributes to ischemic damage of the optic nerve and/or retinal ganglion cells, which plays a prominent role in the progression of glaucoma^38–41^. We postulated that reduction in ocular blood flow may also increase the likelihood of developing low VA in children and adolescents. Therefore, we found that there were significant positive associations between higher prevalence of low VA and elevated BP levels. However, all participants in our study were adolescents aged between 13–18 years, and thus the low vision is always reversible and non-organic. Interestingly, although the increase in SBP and PP was associated with low VA in adolescent males, this association was not obvious in adolescent females. The reason behind these differences in sex was unknown. We hypothesized that the weight of each risk factor for low VA among males and females was different. For example, in females, a positive association between low VA and BP components perhaps also exists, but at a much weaker level than that in males. There may also be other risk factors with greater weight which influenced the low VA in females. Additionally, higher prevalence of low VA was significantly associated with higher PP in females using multivariable logistic regression analyses in 2014 only; however, when PP values were categorized by 10 mm Hg increments, the dose-dependent association between the prevalence of low VA and the levels of PP was not observed. These data supported our hypothesis that the association with low VA still exists in females, albeit at a weaker level. Further studies are needed to better understand the mechanisms and differences in the role of sex and its relationship with VA in adolescent students.

One of the strengths of this study includes the recruitment of three large samples of students collected using the same protocol over a time period of 10 years from Liaoning Province, China. Additionally, we utilized data from 2005, 2010 and 2014, rather than a single survey, to explore the associations between the prevalence of low VA with BP components, thus avoiding accidental results. However, there are some limitations of our study in light of these results. Firstly, the questionnaire was not conducted for all students in 2005, and only the participants who undertook the questionnaire were included in the fully-adjusted model for multivariable logistic regression analyses. Secondly, although we have adjusted for many covariates in our analysis, a few related factors of low VA, such as hereditary traits, time of Internet use and television viewing were not included. Thirdly, the study was based on 3 cross-sectional surveys, as we know cross-sectional study can not determine temporal association. Our finding of BP components as related factors in males requires further verification in longitudinal cohort studies. Lastly, our findings were obtained from school-age adolescents of Han nationality in Northeast China, and therefore are inadequate for the generalization of China on the whole. Thus, further validation of studies which incorporate other regions would better depict the results of a nationwide population.

In conclusion, there are increasing trends of low VA among adolescents in the last decade in Northeast China. Higher prevalence of low VA is significantly associated with higher SBP and PP in adolescent males. Furthermore, there is a dose-dependent association between the prevalence of low VA and an increase in the levels of PP.

## Electronic supplementary material

Supplementary Information
