# Supplementary material for: Elevated blood pressure is associated with higher prevalence of low visual acuity among adolescent males in Northeast China
Source: Sci Rep. 2017 Nov 22;7:15990. doi: 10.1038/s41598-017-14252-9 (PMC5700107; doi:10.1038/s41598-017-14252-9)
Supplement: Supplementary file 1 — Supplementary Information [file 41598_2017_14252_MOESM1_ESM.pdf]

Elevated blood pressure is associated with higher prevalence of low visual acuity among adolescent males in Northeast China

Miaomiao Zhao[1, 2, 3], Wei Wang[1, 2], Han Yu[1, 2], Yunsheng Ma[3], Liqiang Zheng[4], Lijuan Zhang[1, 2], Guiping Wu[5], Yingxian Sun[6*], Jue Li[1, 2*]

1. Key Laboratory of Arrhythmias of Ministry of Education of China, Tongji University School of Medicine, Shanghai, China

2. Institute of Clinical Epidemiology, Tongji University School of Medicine, Shanghai, China

3. Division of Preventive and Behavioral Medicine, Department of Medicine, University of Massachusetts Medical School, Worcester, MA, USA

4. Department of Clinical Epidemiology, Library, Shengjing Hospital of China Medical University, Shenyang, Liaoning, China

5. Department of Cardiology, the Second Hospital Affiliated to Shenyang Medical College, Shenyang, Liaoning, China

6. Department of Cardiology, the First Hospital of China Medical University, Shenyang, Liaoning, China

Corresponding author:

Yingxian Sun

Department of Cardiology, the First Hospital of China Medical University, 155 Nanjing North Street, Heping District, Shenyang, Liaoning 110001, China

Tel: +86-24-83282688

Fax: +86-24-83282346

E-mail: yxsun@cmu.edu.cn

Jue Li

Key Laboratory of Arrhythmias of Ministry of Education of China, Tongji University School of Medicine, 1239 Siping Road, Shanghai 200092, China

Tel: +86-21-65986735

Fax: +86-21-65980448

E-mail: mmz_1989@163.com

Supplementary Table S1. Sample size by age, gender, and region of habitation

	2005		2010		2014	
**Age**	**Urban**	**Rural**	**Urban**	**Rural**	**Urban**	**Rural**
**Boys**						
13	791	797	744	808	721	743
14	802	812	741	826	749	742
15	807	790	815	797	686	696
16	813	814	805	815	722	698
17	806	795	802	823	710	719
18	810	820	793	803	669	616
Total	4829	4828	4700	4872	4257	4214
**Girls**						
13	783	813	740	813	693	744
14	802	774	763	812	714	709
15	806	811	821	804	679	708
16	817	775	826	818	700	705
17	808	797	806	805	697	764
18	811	811	798	795	662	636
Total	4827	4781	4754	4847	4145	4266

Supplementary Table S2. Multiple logistic regression analyses of low VA with blood pressure components and associated factors

Supplementary Table S2.1.1. Multiple logistic regression analyses of low VA with SBP and associated factors in Model 1

	**2005**		**2010**		**2014**	
**Characteristics**	**OR (95% CI)**	***P* value**	**OR (95% CI)**	***P* value**	**OR (95% CI)**	***P* value**
**Males**						
Age, year	1.372(1.333-1.413)	＜0.001	1.246(1.214-1.280)	＜0.001	1.183(1.152-1.216)	＜0.001
Urban, (%)	1.557(1.419-1.709)	＜0.001	1.730(1.586-1.886)	＜0.001	2.028(1.852-2.220)	＜0.001
SBP, mm Hg	1.004(1.000-1.008)	0.031	1.002(0.999-1.006)	0.227	1.010(1.005-1.014)	＜0.001
**Females**						
Age, year	1.382(1.346-1.419)	＜0.001	1.273(1.242-1.305)	＜0.001	1.164(1.134-1.195)	＜0.001
Urban, (%)	1.559(1.429-1.701)	＜0.001	1.804 (1.660-1.961)	＜0.001	1.773(1.625-1.934)	＜0.001
SBP, mm Hg	1.001(0.997-1.005)	0.741	1.001(0.998-1.005)	0.494	1.003(0.999-1.008)	0.115

Abbreviations: VA, visual acuity; SBP, systolic blood pressure.

Supplementary Table S2.1.2. Multiple logistic regression analyses of low VA with DBP and associated factors in Model 1

	**2005**		**2010**		**2014**	
**Characteristics**	**OR (95% CI)**	***P* value**	**OR (95% CI)**	***P* value**	**OR (95% CI)**	***P* value**
**Males**						
Age, year	1.391(1.352-1.432)	＜0.001	1.256(1.224-1.289)	＜0.001	1.198(1.166-1.231)	＜0.001
Urban, (%)	1.567(1.428-1.720)	＜0.001	1.725(1.582-1.881)	＜0.001	2.069(1.891-2.264)	＜0.001
DBP, mm Hg	0.996(0.990-1.001)	0.120	0.996(0.992-1.000)	0.044	1.000(0.995-1.006)	0.912
**Females**						
Age, year	1.384(1.348-1.421)	＜0.001	1.274(1.242-1.306)	＜0.001	1.169(1.139-1.200)	＜0.001
Urban, (%)	1.558(1.428-1.701)	＜0.001	1.801(1.658-1.957)	＜0.001	1.780 (1.631-1.942)	＜0.001
DBP, mm Hg	0.999(0.993-1.004)	0.621	1.001(0.997-1.005)	0.689	0.993(0.988-0.998)	0.009

Abbreviations: VA, visual acuity; DBP, diastolic blood pressure.

Supplementary Table S2.1.3. Multiple logistic regression analyses of low VA with PP and associated factors in Model 1

	**2005**		**2010**		**2014**	
**Characteristics**	**OR (95% CI)**	***P* value**	**OR (95% CI)**	***P* value**	**OR (95% CI)**	***P* value**
**Males**						
Age, year	1.373(1.335-1.413)	＜0.001	1.246(1.214-1.279)	＜0.001	1.194(1.162-1.226)	＜0.001
Urban, (%)	1.566(1.427-1.719)	＜0.001	1.737(1.593-1.894)	＜0.001	2.026(1.851-2.219)	＜0.001
PP, mm Hg	1.010(1.005-1.014)	＜0.001	1.006(1.002-1.010)	0.001	1.011(1.006-1.016)	＜0.001
**Females**						
Age, year	1.382(1.346-1.419)	＜0.001	1.274(1.243-1.306)	＜0.001	1.162(1.133-1.193)	＜0.001
Urban, (%)	1.560(1.430-1.703)	＜0.001	1.802(1.658-1.958)	＜0.001	1.767(1.619-1.928)	＜0.001
PP, mm Hg	1.003(0.997-1.008)	0.350	1.001(0.997-1.005)	0.701	1.013(1.007-1.018)	＜0.001

Abbreviations: VA, visual acuity; PP, pulse pressure.

Supplementary Table S2.2.1. Multiple logistic regression analyses of low VA with SBP and associated factors in Model 2

	2005		2010		2014	
**Characteristics**	**OR (95% CI)**	***P* value**	**OR (95% CI)**	***P* value**	**OR (95% CI)**	***P* value**
**Males**						
Age, year	1.371(1.331-1.412)	＜0.001	1.252(1.219-1.286)	＜0.001	1.185(1.153-1.218)	＜0.001
Urban, (%)	1.553(1.415-1.706)	＜0.001	1.755(1.608-1.914)	＜0.001	2.040(1.863-2.234)	＜0.001
BMI, kg/m^2	1.003(0.988-1.019)	0.662	0.981(0.968-0.995)	0.007	0.987(0.975-0.999)	0.030
SBP, mm Hg	1.004(1.000-1.008)	0.048	1.004(1.000-1.007)	0.056	1.011 (1.007-1.016)	＜0.001
**Females**						
Age, year	1.385(1.348-1.423)	＜0.001	1.273(1.241-1.305)	＜0.001	1.160(1.130-1.191)	＜0.001
Urban, (%)	1.560(1.430-1.703)	＜0.001	1.804(1.660-1.961)	＜0.001	1.774(1.626-1.936)	＜0.001
BMI, kg/m^2	0.993(0.977-1.009)	0.409	1.000(0.986-1.015)	0.980	1.015(1.001-1.030)	0.036
SBP, mm Hg	1.001(0.997-1.005)	0.618	1.001(0.997-1.005)	0.511	1.002(0.998-1.007)	0.320

Abbreviations: VA, visual acuity; SBP, systolic blood pressure; BMI, body mass index.

Supplementary Table S2.2.2. Multiple logistic regression analyses of low VA with DBP and associated factors in Model 2

	2005		2010		2014	
**Characteristics**	**OR (95% CI)**	***P* value**	**OR (95% CI)**	***P* value**	**OR (95% CI)**	***P* value**
**Males**						
Age, year	1.387(1.347-1.428)	＜0.001	1.262(1.229-1.296)	＜0.001	1.199(1.167-1.232)	＜0.001
Urban, (%)	1.557(1.418-1.710)	＜0.001	1.741(1.596-1.899)	＜0.001	2.075(1.896-2.271)	＜0.001
BMI, kg/m^2	1.010(0.995-1.025)	0.194	0.987(0.974-1.000)	0.043	0.996(0.984-1.008)	0.493
DBP, mm Hg	0.995(0.990-1.001)	0.081	0.996(0.992-1.001)	0.086	1.001(0.995-1.006)	0.809
**Females**						
Age, year	1.386(1.349-1.424)	＜0.001	1.273(1.242-1.306)	＜0.001	1.163(1.133-1.194)	＜0.001
Urban, (%)	1.559(1.429-1.701)	＜0.001	1.801(1.657-1.956)	＜0.001	1.781(1.632-1.943)	＜0.001
BMI, kg/m^2	0.995(0.979-1.011)	0.508	1.001(0.987-1.016)	0.894	1.021(1.007-1.035)	0.004
DBP, mm Hg	0.999(0.994-1.004)	0.708	1.001(0.997-1.005)	0.707	0.992(0.987-0.997)	0.003

Abbreviations: VA, visual acuity; DBP, diastolic blood pressure; BMI, body mass index.

Supplementary Table S2.2.3. Multiple logistic regression analyses of low VA with PP and associated factors in Model 2

	2005		2010		2014	
**Characteristics**	**OR (95% CI)**	***P* value**	**OR (95% CI)**	***P* value**	**OR (95% CI)**	***P* value**
**Males**						
Age, year	1.372(1.333-1.413)	＜0.001	1.254(1.222-1.288)	＜0.001	1.196(1.164-1.229)	＜0.001
Urban, (%)	1.564(1.424-1.718)	＜0.001	1.762(1.615-1.922)	＜0.001	2.036(1.859-2.230)	＜0.001
BMI, kg/m^2	1.002(0.987-1.017)	0.826	0.981(0.968-0.994)	0.005	0.991(0.980-1.003)	0.142
PP, mm Hg	1.009(1.005-1.014)	＜0.001	1.007(1.003-1.011)	＜0.001	1.012(1.007-1.016)	＜0.001
**Females**						
Age, year	1.385(1.348-1.423)	＜0.001	1.274(1.242-1.306)	＜0.001	1.159(1.129-1.189)	＜0.001
Urban, (%)	1.561(1.431-1.704)	＜0.001	1.801(1.658-1.957)	＜0.001	1.767(1.620-1.929)	＜0.001
BMI, kg/m^2	0.993(0.977-1.009)	0.394	1.001(0.987-1.016)	0.886	1.012(0.998-1.027)	0.082
PP, mm Hg	1.003(0.997-1.008)	0.303	1.001(0.997-1.005)	0.718	1.012(1.006-1.017)	＜0.001

Abbreviations: VA, visual acuity; PP, pulse pressure; BMI, body mass index.

Supplementary Table S2.3.1. Multiple logistic regression analyses of low VA with SBP and associated factors in Model 3

	**2005**		**2010**		**2014**	
**Characteristics**	**OR (95% CI)**	***P* value**	**OR (95% CI)**	***P* value**	**OR (95% CI)**	***P* value**
**Males**						
Age, year	1.411(1.332-1.496)	＜0.001	1.223(1.188-1.259)	＜0.001	1.165(1.131-1.200)	＜0.001
Urban, (%)	1.519(1.273-1.813)	＜0.001	1.735(1.587-1.896)	＜0.001	1.973(1.796-2.168)	＜0.001
BMI, kg/m^2	0.997(0.969-1.026)	0.853	0.977(0.964-0.991)	0.001	0.983(0.971-0.996)	0.008
SBP, mm Hg	1.008(1.001-1.016)	0.028	1.005(1.001-1.009)	0.017	1.011(1.006-1.016)	＜0.001
Sleep duration, h/day	0.960(0.883-1.044)	0.343	0.962(0.919-1.007)	0.099	0.946(0.903-0.991)	0.018
Outdoor activity time, h/day	0.927(0.796-1.078)	0.324	0.856(0.791-0.928)	＜0.001	0.852(0.787-0.922)	＜0.001
Homework time, h/day	1.121(1.031-1.219)	0.008	1.050(1.003-1.099)	0.035	1.062(1.015-1.112)	0.010
**Females**						
Age, year	1.389(1.316-1.465)	＜0.001	1.261(1.226-1.297)	＜0.001	1.130(1.098-1.163)	＜0.001
Urban, (%)	1.510(1.279-1.783)	＜0.001	1.737(1.596-1.891)	＜0.001	1.757(1.605-1.924	＜0.001
BMI, kg/m^2	0.999(0.968-1.031)	0.944	1.000(0.985-1.015)	0.975	1.013(0.998-1.028)	0.079
SBP, mm Hg	1.005(0.998-1.013)	0.168	1.001(0.997-1.005)	0.490	1.002(0.997-1.006)	0.502
Sleep duration, h/day	0.963(0.881-1.053)	0.408	0.962(0.917-1.009)	0.110	0.891(0.849-0.936)	＜0.001
Outdoor activity time, h/day	0.830(0.703-0.982)	0.029	0.969(0.890-1.056)	0.474	0.804(0.737-0.879)	＜0.001
Homework time, h/day	1.043(0.960-1.134)	0.322	1.049(1.003-1.096)	0.036	1.081(1.031-1.132)	0.001

Abbreviations: VA, visual acuity; SBP, systolic blood pressure; BMI, body mass index.

Data in 2005 available for participants who undertook questionnaire.

Supplementary Table S2.3.2. Multiple logistic regression analyses of low VA with DBP and associated factors in Model 3

	**2005**		**2010**		**2014**	
**Characteristics**	**OR (95% CI)**	***P* value**	**OR (95% CI)**	***P* value**	**OR (95% CI)**	***P* value**
**Males**						
Age, year	1.436(1.354-1.522)	＜0.001	1.233(1.198-1.270)	＜0.001	1.179(1.145-1.214)	＜0.001
Urban, (%)	1.521(1.274-1.815)	＜0.001	1.717(1.572-1.876)	＜0.001	2.009(1.829-2.206)	＜0.001
BMI, kg/m^2	1.009(0.982-1.037)	0.501	0.983(0.970-0.997)	0.014	0.993(0.981-1.005)	0.229
DBP, mm Hg	0.995(0.985-1.005)	0.333	0.997(0.992-1.001)	0.105	1.000(0.994-1.005)	0.905
Sleep duration, h/day	0.960(0.883-1.043)	0.336	0.961(0.917-1.006)	0.087	0.944(0.901-0.989)	0.015
Outdoor activity time, h/day	0.933(0.802-1.086)	0.373	0.856(0.790-0.927)	＜0.001	0.849(0.785-0.919)	＜0.001
Homework time, h/day	1.125(1.034-1.223)	0.006	1.051(1.004-1.100)	0.032	1.063(1.015-1.112)	0.009
**Females**						
Age, year	1.392(1.319-1.469)	＜0.001	1.261(1.226-1.297)	＜0.001	1.133(1.101-1.166)	＜0.001
Urban, (%)	1.500(1.271-1.771)	＜0.001	1.734(1.593-1.888)	＜0.001	1.765(1.613-1.933)	＜0.001
BMI, kg/m^2	1.005(0.974-1.036)	0.773	1.000(0.986-1.015)	0.954	1.018(1.004-1.033)	0.013
DBP, mm Hg	1.000(0.990-1.010)	0.969	1.001(0.997-1.006)	0.569	0.991(0.986-0.996)	0.001
Sleep duration, h/day	0.961(0.880-1.051)	0.383	0.963(0.918-1.009)	0.116	0.891(0.849-0.936)	＜0.001
Outdoor activity time, h/day	0.830(0.703-0.982)	0.029	0.969(0.889-1.055)	0.463	0.803(0.735-0.877)	＜0.001
Homework time, h/day	1.041(0.958-1.132)	0.340	1.049(1.003-1.097)	0.035	1.079(1.030-1.130)	0.001

Abbreviations: VA, visual acuity; DBP, diastolic blood pressure; BMI, body mass index.

Data in 2005 available for participants who undertook questionnaire.

Supplementary Table S2.3.3. Multiple logistic regression analyses of low VA with PP and associated factors in Model 3

	**2005**		**2010**		**2014**	
**Characteristics**	**OR (95% CI)**	***P* value**	**OR (95% CI)**	***P* value**	**OR (95% CI)**	***P* value**
**Males**						
Age, year	1.421(1.342-1.505)	＜0.001	1.225(1.190-1.261)	＜0.001	1.175(1.142-1.210)	＜0.001
Urban, (%)	1.532(1.283-1.829)	＜0.001	1.739(1.591-1.901)	＜0.001	1.965(1.788-2.159)	＜0.001
BMI, kg/m^2	0.996(0.968-1.024)	0.759	0.977(0.964-0.991)	0.001	0.987(0.975-0.999)	0.039
PP, mm Hg	1.016(1.007-1.025)	＜0.001	1.008(1.004-1.012)	＜0.001	1.012(1.007-1.017)	＜0.001
Sleep duration, h/day	0.960(0.883-1.044)	0.340	0.959(0.916-1.004)	0.074	0.944(0.901-0.989)	0.015
Outdoor activity time, h/day	0.930(0.799-1.083)	0.350	0.857(0.791-0.928)	＜0.001	0.851(0.786-0.921)	＜0.001
Homework time, h/day	1.120(1.030-1.218)	0.008	1.048(1.002-1.097)	0.042	1.062(1.015-1.112)	0.010
**Females**						
Age, year	1.391(1.318-1.467)	＜0.001	1.261(1.226-1.298)	＜0.001	1.129(1.097-1.162)	＜0.001
Urban, (%)	1.509(1.278-1.782)	＜0.001	1.733(1.592-1.886)	＜0.001	1.750(1.598-1.916)	＜0.001
BMI, kg/m^2	1.000(0.970-1.032)	0.977	1.001(0.986-1.016)	0.909	1.010(0.995-1.024)	0.188
PP, mm Hg	1.009(0.999-1.019)	0.066	1.000(0.996-1.005)	0.839	1.012(1.007-1.018)	＜0.001
Sleep duration, h/day	0.962(0.880-1.051)	0.387	0.962(0.918-1.009)	0.113	0.893(0.850-0.937)	＜0.001
Outdoor activity time, h/day	0.830(0.702-0.981)	0.029	0.969(0.890-1.056)	0.473	0.803(0.735-0.877)	＜0.001
Homework time, h/day	1.043(0.960-1.134)	0.321	1.048(1.003-1.096)	0.037	1.079(1.030-1.130)	0.001

Abbreviations: VA, visual acuity; PP, pulse pressure; BMI, body mass index.

Data in 2005 available for participants who undertook questionnaire.
